# The Effect of Explicit Suicide Language in Engagement With a Suicide Prevention Search Page Help-Seeking Prompt: Nonrandomized Trial

**DOI:** 10.2196/50283

**Published:** 2024-03-19

**Authors:** Sandersan Onie, Susanne Oliver Armstrong, Natasha Josifovski, Patrick Berlinquette, Nicola Livingstone, Sarah Holland, Coco Finemore, Nyree Gale, Emma Elder, George Laggis, Cassandra Heffernan, Adam Theobald, Michelle Torok, Fiona Shand, Mark Larsen

**Affiliations:** 1 Black Dog Institute University of New South Wales Sydney Australia; 2 Faculty of Psychology Universitas Airlangga Surabaya Indonesia; 3 BERLIN SEM New York, NY United States

**Keywords:** suicide, suicide prevention, Google, Google Ads, internet search, explicit wording, mental health, suicidal, advertisement, advertisements, messaging, prevention signage, campaign, campaigns, distress, engagement, prompt, prompts, information seeking, help seeking, searching, search

## Abstract

**Background:**

Given that signage, messaging, and advertisements (ads) are the gateway to many interventions in suicide prevention, it is important that we understand what type of messaging works best for whom.

**Objective:**

We investigated whether explicitly mentioning suicide increases engagement using internet ads by investigating engagement with campaigns with different categories of keywords searched, which may reflect different cognitive states.

**Methods:**

We ran a 2-arm study Australia-wide, with or without ads featuring explicit suicide wording. We analyzed whether there were differences in engagement for campaigns with explicit and nonexplicit ads for low-risk (distressed but not explicitly suicidal), high-risk (explicitly suicidal), and help-seeking for suicide keywords.

**Results:**

Our analyses revealed that having explicit wording has opposite effects, depending on the search terms used: explicit wording reduced the engagement rate for individuals searching for low-risk keywords but increased engagement for those using high-risk keywords.

**Conclusions:**

The findings suggest that individuals who are aware of their suicidality respond better to campaigns that explicitly use the word “suicide.” We found that individuals who search for low-risk keywords also respond to explicit ads, suggesting that some individuals who are experiencing suicidality search for low-risk keywords.

## Introduction

Over the past decade, there has been a growth in the different types of interventions for people contemplating suicide. For example, apps can help people keep themselves safe during a suicidal crisis [1]; phone booths are installed at frequently used locations, which give rapid access to a suicide hotline [2]; or an online banner containing a suicide hotline number may appear if individuals search for suicide-related terms using a search engine [3]. Despite these help-seeking pathways, recent research has shown that less than half of individuals contemplating suicide seek professional help before a suicide attempt [4]. Thus, in parallel to developing new interventions, it may be important to understand how best to promote help seeking and access to help for individuals contemplating suicide for existing services and products.

Help-seeking prompts may function as a gateway to these interventions and come in various forms. In this case, a help-seeking prompt refers to any media encouraging service use. For example, a person may prompt an individual to see their general practitioner (GP) for their mental health, a description on an app store may prompt them to download a suicide prevention app, signage at frequently used locations may point them toward a phone booth, or text on an internet search page may encourage them to call a hotline. All these help-seeking prompts play an important role in being the first point of engagement for the individual, introducing the intervention and promoting its use. If the advertisement (ad) cannot encourage the individual to engage with the intervention, it cannot fulfil its role.

One way we can investigate how different types of prompt messaging affect engagement is by using internet search ads. Previous studies have shown that individuals may search for suicide-related terms on the internet before a suicide attempt [5], that search volumes for a particular region correspond to the suicide rate for that region [6], and that internet searches are used to seek help [7]. For example, an individual may use the internet to find the closest crisis center, a local psychiatrist, or an app to help manage their suicidal thoughts. Searching for “suicide help” yields over 1 billion results on Google, and previous studies have shown a high frequency of searches, with over 120,000 searches over a 19-day study period for suicide- and distress-related keywords in Australia alone [7]. Thus, the search page may be an ideal place to intervene and investigate what messaging is most effective for individuals contemplating suicide.

Internet search ads are triggered when keywords from a prepared list are used, presenting an ad at the top of the search results that links to a relevant page or intervention. Internet search ads also offer the ability to assess how different types of messaging perform with people in different cognitive states, reflected by their search terms. For example, individuals searching for keywords associated with suicide but not explicitly communicating suicidality (eg, loneliness, hopelessness) may engage differently with a particular ad wording than individuals searching for keywords explicitly indicating suicidality or seeking help. This allows us to assess what messaging is best for different risk or distress levels.

The reach and effectiveness of these ads are measured with the impressions (how many times an ad is shown), clicks (the number of clicks on the ad), and conversions (specific behaviors performed on the linked website). Engagement is specifically measured with the click rate (the proportion of individuals who saw the ad and then clicked on it), the conversion rate (the proportion of individuals who clicked on the ad and then engaged with the website), and the total conversion rate (the proportion of individuals who saw the ad and then engaged with the website) [7].

A previous study has highlighted that one major discussion area regarding communicating with an individual contemplating suicide is the explicit use of the word “suicide” in the ad [8]. In one component of this study, lived experience advisors indicated that the use of the word “suicide” in ads might alienate some individuals who may indeed be experiencing thoughts of suicide but may not recognize, acknowledge, or identify their thoughts as being of suicide. The advisors elaborated that by not using the word “suicide,” we may be able to reach individuals at a precrisis phase for early intervention.

Conversely, other lived experience advisors from the same study communicated that it is imperative to be clear on the subject matter by using the word “suicide.” These advisors communicated that by being explicit, we can overcome the stigma associated with the word and the individual contemplating suicide may be more likely to engage with the service as it is specific to their needs or current situation. This is further supported by contemporary suicide first aid programs, which encourage the explicit use of the word “suicide” for the same reason [9]. Furthermore, all lived experience advisors highlighted the importance of understanding what wording is effective for different suicide risk levels to maximize engagement. For example, nonexplicit wording may be particularly effective for individuals contemplating suicide but not in a suicidal crisis, as they may not identify their thoughts being that of suicide—and vice versa.

In this study, we sought to compare engagement with two internet ad campaigns, one with explicit suicide wording in its ad and the other with nonexplicit suicide wording. We also investigated whether the pattern of engagement differed by the type of keyword searched (low risk, high risk, help seeking, or means specific). In addition, we examined engagement by gender, age, and time of day.

First, we hypothesized that the campaign with explicit wording related to suicide would perform better for individuals searching for high-risk, help-seeking, or means-specific keywords. Given that these individuals are explicitly experiencing suicidal ideation and can identify and communicate it, they may respond better to an ad that is explicit in what issue it is addressing. Second, we hypothesized that for individuals searching for low risk-keywords, nonexplicit wording would have higher engagement as the explicit wording may alienate individuals who do not identify as having suicidal thoughts. Third, given that the key manipulation is in the ad’s wording and not the linked web pages, we hypothesized that we should see this increased engagement in the click rate but not the conversion rate (and, as such, also see increased engagement in the total conversion rate).

## Methods

### Study Design

The study used a 2-arm quasi-experimental design (explicit vs nonexplicit wording) with 4 initial pathways (individuals searching for different types of keywords: low risk, high risk, help seeking, and means specific; these categories are described in more detail later).

### Participants

Individuals over the age of 18 years and currently residing in Australia were included in the study. Google infers the age and gender of individuals through multiple sources of information, primarily the age and gender inputted when creating a Google account, as well as past browsing history (eg, websites visited and engagements) collected through website cookies.

### Ethical Considerations

The University of New South Wales Human Research Ethics Committee approved this study (HC210827).

### Intervention

**Figure 1:**
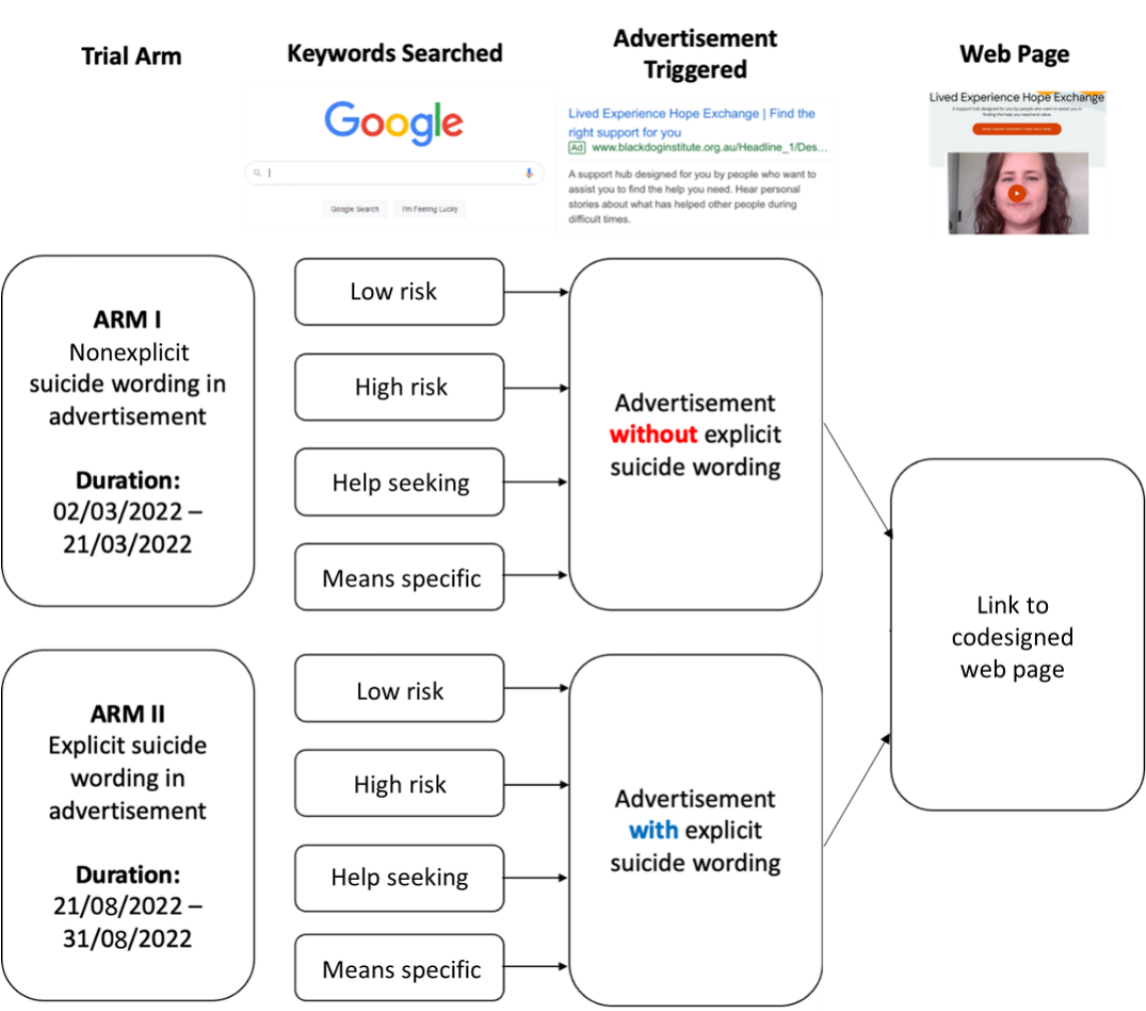
Schematic of Google Ads campaign.

The first arm used ads without explicit suicide wording and ran from March 2 to March 21, 2022. The second arm used ads with explicit suicide wording and ran from August 21 to August 31, 2022. The first arm was run as part of another larger study pertaining to the effectiveness of Google Ads campaigns in reaching individuals thinking of suicide and has been reported elsewhere [7]. The 2 arms of the trial were run sequentially, not concurrently. Thus, there was no randomization present. Full details of the keyword generation, ad and landing page codesign process, and content of the landing pages and linked pages can be found elsewhere [8]. A schematic of the campaign can be found in Figure 1.

### Keywords

Together with lived experience advisors, researchers, and a Google Ads agent, we generated 4 lists of keywords: low-risk keywords, which included keywords people are likely to search for when in distress or situations associated with suicide, without explicitly mentioning suicide (eg, “feeling so alone,” “debt”); high-risk keywords, which included keywords explicitly communicating suicidal ideation or help seeking (eg, “I want to die”); help-seeking keywords, which included keywords explicitly searching for help for suicidal thoughts (eg, “suicide help”); and means-specific keywords, which were related to searching or using specific means [10]. When keywords are entered into Google Ads, the ad is also triggered when semantically similar search terms are entered; thus, the total number of keywords that trigger the ad is substantially larger than what is inputted.

### Advertisements

The ads were codesigned alongside a group of lived experience advisors and investigators. The codesign process yielded 6 ads, 3 (50%) explicit suicide wording and 3 (50%) without explicit suicide wording, while controlling as much as possible for other content. Each ad was as closely matched as possible to its counterpart; that is, the first ad in the explicit condition had the same messaging and content as the first ad in the nonexplicit condition, save for explicit suicide wording in the first sentence. We developed more than 1 ad for each condition to allow our findings to be more generalizable and to reflect larger themes found in our codesign process [8] rather than specific wording.

All the ads across both conditions were controlled for the number of characters and number of words, with a range of 38-43 words and 215-234 characters. The absolute difference in the character and word count between each ad and its counterpart was between 1 and 5 characters and 1 and 3 words, respectively.

The codesigned text for the nonexplicit ads is specified next. All character counts include spaces.

Lived Experience Hope Exchange. Find the right support for you. A support hub designed for you by people who want to assist you to find the help you need. Hear personal stories about what has helped other people during difficult times. (237 characters, 41 words)

Looking for some support? Designed with Lived Experience. Our Hope Exchange has been designed by people who may understand how you're feeling. There are lots of ways to seek help. We want to find the right one for you. (220 characters, 39 words)

Find the right support for you. Lived Experience Hope Exchange. We want to help you to find the help that you need and value during challenging times. Hear stories and advice from people who may have felt the way you're feeling now. (234 characters, 42 words)

The matched text for the explicit wording ads was as follows:

Are you feeling suicidal? Lived Experience Hope Exchange. Support designed for you. Designed by people who want to assist you to find the help you need. Hear personal stories about what has helped other people during difficult times. (235 characters, 38 words)

Help for suicidal thoughts. Looking for some support? Designed with Lived Experience. Designed by people who may understand how you're feeling. There are lots of ways to seek help. We want to find the right one for you. (221 characters, 38 words)

Dealing with suicidal thoughts. Find the right support for you. Lived Experience Hope Exchange. We want to help you to find the help that you need and value. Hear stories and advice from people who may have felt the way you're feeling now. (241 characters, 43 words)

In each campaign condition, ads were shown to users independently of which category of keywords were searched. When an ad was triggered, 1 of 3 ads in that condition would be randomly shown, resulting in equal presentations across the study.

### Landing Page

In collaboration with lived experience advisors, we codesigned a series of landing pages containing lived experience stories, calming and distracting activities, and links to support services and hotlines with descriptions of what the individual will likely experience when engaging in these services. Details of the pages can be found elsewhere [8].

### Outcomes

Data on impressions, clicks, the click rate (clicks/impressions), conversions, the conversion rate (conversions/clicks), the cost per click, and the cost per conversion were extracted from Google Ads in a deidentified, aggregated form. The total conversion rate was manually calculated (conversions/impressions). Currently, the total volume of searches for each category is not available through Google Ads.

The primary outcome was the click rate (engagement with the ad) as our manipulation was on the search page rather than on the landing page. Our secondary outcomes were the total conversion rate (total engagement with the campaign, that is, all things being equal, the conversion rate per impression) and the conversion rate (engagement with the landing page).

Conversions contained behaviors the investigators, the lived experience advisors, and the collaborative team considered positive. Triggering any of these conditions was considered a conversion, including:

- Clicking the Get Help button to see available support services
- Downloading any file pertaining to the modules to help de-escalate a crisis or for self-help for suicidality
- Clicking on a link to call a support service
- Spending more than 2 minutes on the website that was designed to promote help seeking and de-escalate crises, as an indication that the individual was engaging with content on the website

### Statistical Analyses

In the main analysis, each combination of outcome metric (click rate, conversion rate, and total conversation rate) and keyword type (high risk, low risk, and help seeking) was considered separately. The outcome metric rates associated with the explicit and nonexplicit wording were compared using an incidence rate ratio (IRR) from the *rateratio* function in the *fmsb* package in R (R Foundation for Statistical Computing), which calculates the exact mid-p double-sided *P* value and calculates the CI using the exact Poisson method [11,12].

Interaction terms were considered if at least 1 significant difference between explicit and nonexplicit wording was identified for an outcome measure. In this case, a keyword category with a significant difference due to wording was compared to the other keyword categories. To test the interactions, we first calculated a difference term between the explicit and nonexplicit conditions for a single keyword type. For example, we calculated the difference term for the click rate and low-risk keywords as follows:



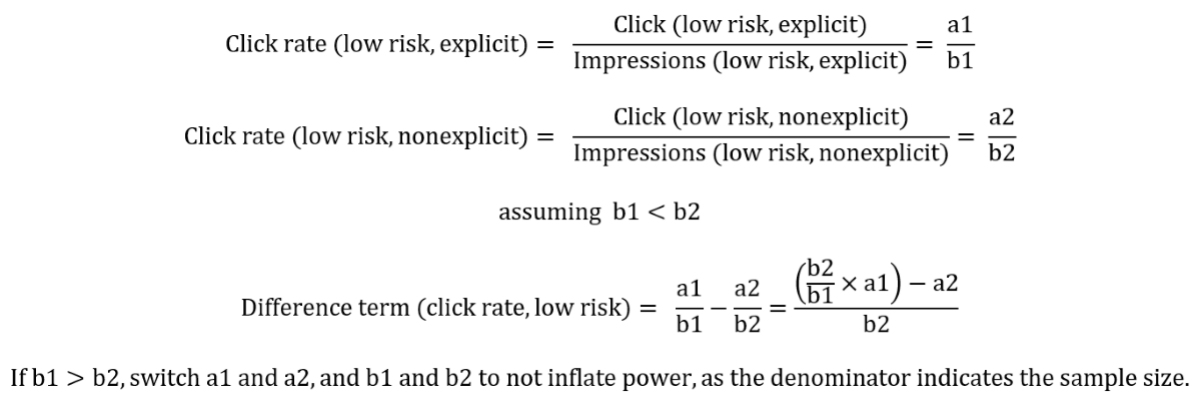



This difference term was compared with the other keyword difference terms using the *ratedifference* function in the *fmsb* package in R, which uses a chi-squared test to test for a significant incidence rate difference (IRD) [12]. Thus, this analysis assessed interaction by assessing a difference of a difference.

Where a significant difference of differences (ie, an interaction) was identified in the outcomes, post hoc tests were conducted to determine whether there were underlying differences in the relevant outcome metrics for the explicit or nonexplicit wording conditions or both.

## Results

### Campaign Metrics

A total of 153,768 impressions, 7263 clicks, and 1657 conversions were achieved during the study periods. The engagement metrics, reported by trial condition, age, and gender, are reported in Table 1.

Due to the exceptionally low numbers in the means-specific group (n=11, 0.01%, impressions; n=1, 0.01%, click; and 0 conversions), these campaigns were excluded from subsequent analyses.

**Table 1:** Engagement metrics by trial condition, age, and gender.

Trial condition, gender, and age (years)		Impressions (N=153,768), n (%)	Clicks (N=7263), n (%)	Click rate, %	Conversions (N=1657), n (%)	Conversion rate, %	Total conversion rate, %
**Nonexplicit trial, gender male**							
	18-24	6191 (4.03)	308 (4.24)	4.97	39 (2.35)	12.66	0.63
	25-34	6955 (4.52)	261 (3.59)	3.75	52 (3.14)	19.92	0.75
	35-44	6770 (4.40)	313 (4.31)	4.62	77 (4.65)	24.60	1.14
	45-54	6975 (4.54)	398 (5.48)	5.71	122 (7.36)	30.65	1.75
	55-64	4644 (3.02)	257 (3.54)	5.53	93 (5.61)	36.19	2.00
	≥65	2695 (1.75)	169 (2.33)	6.27	36 (2.17)	21.30	1.34
	All ages	34,230 (22.26)	1706 (23.49)	4.98	419 (25.29)	24.56	1.22
**Nonexplicit trial, gender female**							
	18-24	13,243 (8.61)	796 (10.96)	6.01	89 (5.37)	11.18	0.67
	25-34	17,448 (11.35)	708 (9.75)	4.06	135 (8.15)	19.07	0.77
	35-44	17,996 (11.70)	869 (11.96)	4.83	180 (10.86)	20.71	1.00
	45-54	18,963 (12.33)	1013 (13.95)	5.34	311 (18.77)	30.70	1.64
	55-64	12,675 (8.24)	737 (10.15)	5.81	187 (11.29)	25.37	1.48
	≥65	6326 (4.11)	398 (5.48)	6.29	98 (5.91)	24.62	1.55
	All ages	86,651 (56.35)	4521 (62.25)	5.22	1000 (60.35)	22.12	1.15
**Explicit trial, gender male**							
	18-24	1825 (1.19)	62 (0.85)	3.40	4 (0.24)	6.45	0.22
	25-34	2281 (1.48)	62 (0.85)	2.72	21 (1.27)	33.87	0.92
	35-44	2052 (1.33)	64 (0.88)	3.12	13 (0.78)	20.31	0.63
	45-54	1941 (1.26)	76 (1.05)	3.92	19 (1.15)	25.00	0.98
	55-64	1351 (0.88)	54 (0.74)	4.00	15 (0.90)	27.78	1.11
	≥65	841 (0.55)	46 (0.63)	5.47	8 (0.48)	17.39	0.95
	All ages	10,291 (6.69)	364 (5.01)	3.54	80 (4.83)	21.98	0.78
**Explicit trial, gender female**							
	18-24	3436 (2.23)	104 (1.43)	3.03	16 (0.97)	15.38	0.47
	25-34	4474 (2.91)	108 (1.49)	2.41	21 (1.27)	19.44	0.47
	35-44	4489 (2.92)	140 (1.93)	3.12	30 (1.81)	21.43	0.67
	45-54	4824 (3.14)	136 (1.87)	2.82	37 (2.23)	27.21	0.77
	55-64	3381 (2.20)	97 (1.34)	2.87	28 (1.69)	28.87	0.83
	≥65	1992 (1.30)	87 (1.20)	4.37	26 (1.57)	29.89	1.31
	All ages	22,596 (14.69)	672 (9.25)	2.97	158 (9.54)	23.51	0.70
**Combined trial**							
	All ages, male	44,521 (28.95)	2070 (28.50)	4.65	499 (30.11)	24.11	1.12
	All ages, female	109,247 (71.05)	5193 (71.50)	4.75	1158 (69.89)	22.30	1.06

### Click Rate

**Figure 2:**
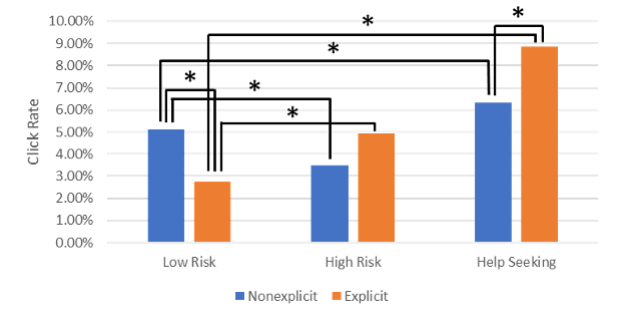
Click rate by keyword type and condition. *P<.05.

The click rate using explicit and nonexplicit wording for each keyword category is shown in Figure 2. A significant difference between explicit and nonexplicit wording for low-risk keywords was found (IRR=1.848, 95% CI 1.718-1.987, *P*<.001), in which there was a higher click rate for nonexplicit versus explicit keywords (5.11% vs 2.77%). For help-seeking keywords, there was a significantly higher click rate for explicit versus nonexplicit keywords (3.50% vs 4.93%; IRR=0.715, 95% CI 0.599-0.854, *P*<.001). A similar pattern was observed for high-risk keywords, with a higher click rate for explicit versus nonexplicit keywords, although this was only marginally nonsignificant (6.33% vs 8.85%; IRR=0.711, 95% CI 0.503-1.005, *P*=.052).

Next, we investigated the presence of 2-way interactions between the 3 groups (low risk and high risk, low risk and help seeking, high risk and help seeking). All 2-way interactions were significant (high risk and help seeking: IRD=0.0109, 95% CI 0.000146-0.0217, *P*=.047; high risk and low risk: IRD=0.0301, 95% CI 0.0301-0.0453, *P*<.001; low risk and help seeking: IRD=0.0472, 95% CI 0.0405-0.0541, *P*<.001).

Post hoc tests revealed a significant difference in the click rate between the low- and high-risk keywords in the explicit condition (2.77% vs 4.93%; IRR=0.5612, 95% CI 0.4144-0.7784, *P*<.001) and the nonexplicit condition (5.11% vs 3.50%; IRR=1.459, 95% CI 1.216-1.766, *P*<.001). Significant differences were also found in the click rate between the low-risk and help-seeking keywords in the explicit condition (2.77% vs 8.85%; IRR=0.312, 95% CI 0.263-0.373, *P*<.001) and the nonexplicit condition (5.11% vs 6.33%; IRR=0.807, 95% CI 0.740-0.881, *P*<.001).

### Conversion Rate

**Figure 3:**
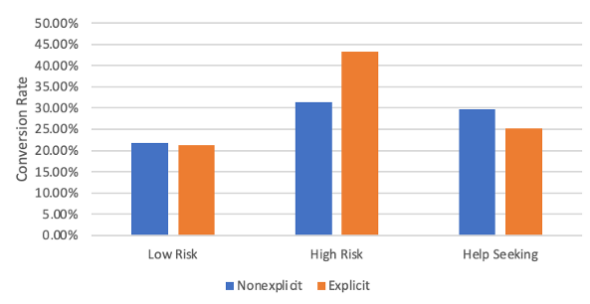
Conversion rate by keyword type and condition.

A graphical representation of the conversion rate data is shown in Figure 3. There were no significant differences between the nonexplicit and explicit conditions (low risk: 21.86% vs. 21.17%; IRR=1.0326, 95% CI 0.882-1.209, *P*=.69; high risk: 31.36% vs 43.18%; IRR=0.726, 95% CI 0.418-1.263, *P*=.25; help seeking: 29.74% vs 25.16%; IRR=1.182, 95% CI 0.835-1.674, *P*=.35). Thus, interaction effects were not explored.

### Total Conversion Rate

**Figure 4:**
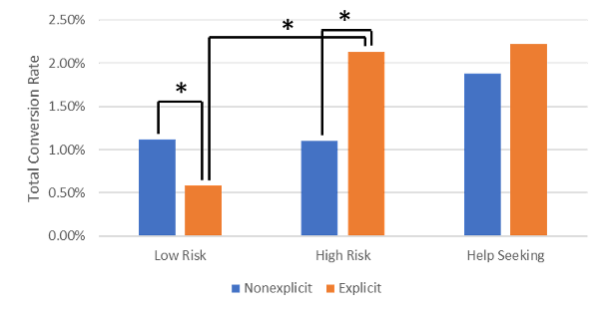
Total conversion rate by keyword type and condition. *P<.05.

A graphical representation of the total conversion rate can be seen in Figure 4. There was a significant difference in the total conversion rate for low-risk keywords (IRR=1.908, 95% CI 1.630-2.234, *P*<.001), in which the nonexplicit wording had a higher rate (1.12% vs 0.59%), and high-risk keywords (IRR=0.846, 95% CI 0.297-0.896, *P*=.02), in which the explicit wording had a higher rate (1.10% vs 2.13%); however, there was not enough evidence to suggest a difference between explicit and nonexplicit wording (1.88% vs 2.23%) when an individual was searching for help-seeking keywords (IRR=0.846, 95% CI 0.597-1.197, *P*=.34).

Possible interactions between the 3 groups (low risk and high risk, low risk and help seeking, high risk and help seeking) were explored. A significant interaction was identified between low-risk and high-risk keywords (IRD=0.00875, 95% CI 0.00613-0.0114, *P*<.001), as well as between low-risk and help-seeking keywords (IRD=0.0156, 95% CI 0.00901-0.0222, *P*<.001). However, there was no significant interaction between high-risk and help-seeking keywords (IRD=–0.00685, 95% CI –0.0141 to 0.000345, *P*=.06).

Post hoc tests revealed a significant difference in the total conversion rate between the low- and high-risk keywords in the explicit condition (0.59% vs 2.13%; IRR=0.275, 95% CI 0.171-0.442, *P*<.001) but not in the nonexplicit condition (1.12% vs 1.10%; IRR=1.017, 95% CI 0.733-1.411, *P*=.92).

A summary of the analysis outcomes can be seen in Table 2.

**Table 2:** Summary of analysis outcomes.

Metric and keyword type		Significant comparisons	Significant interactions	
**Click rate**				
	Low risk	Nonexplicit > explicit	Low risk × high risk Low risk × help seeking High risk × help seeking	
	High risk	Explicit > nonexplicit^a^	Low risk × high risk Low risk × help seeking High risk × help seeking	
	Help seeking	Explicit > nonexplicit	Low risk × high risk Low risk × help seeking High risk × help seeking	
**Conversion rate**				
	Low risk	N/S^b^	—^c^	
	High risk	N/S	—	
	Help seeking	N/S	—	
**Total conversion rate**				
	Low risk	Nonexplicit > explicit	Low risk × high risk Low risk × help seeking	
	High risk	Explicit > nonexplicit	Low risk × high risk Low risk × help seeking	
	Help seeking	N/S	Low risk × high risk Low risk × help seeking	

^a^Marginal significance (*P*<.06).

^b^N/S: not significant.

^c^Not available. This was used when interaction analyses were not conducted due to nonsignificant comparisons.

## Discussion

### Principal Findings

In this study, we compared the impact of explicit and nonexplicit suicide wording in an online ad campaign and webpage targeting those searching for suicide- and distress-related keywords. Analysis of the click rate revealed that for low-risk keywords, nonexplicit wording had a higher click rate; for high-risk keywords, there was marginal evidence that explicit wording had a higher click rate; and for help-seeking keywords, explicit wording had a higher click rate. For the conversion rate, there was no evidence of any differences between conditions. Analysis of the total conversion rate revealed that for low-risk keywords, nonexplicit wording had a higher total conversion rate; for high-risk keywords, explicit wording had a higher total conversion rate; and for help-seeking keywords, there was no evidence of a difference.

Further analysis of the click rate revealed 2-way interactions between low- and high-risk keywords, low-risk and help-seeking keywords, and high-risk and help-seeking keywords, suggesting that the effect of explicit wording in the ad differed among these groups. Further exploratory analysis revealed significant differences between low-risk and high-risk keywords, as well as between low-risk and help-seeking keywords, in the explicit and nonexplicit conditions. These findings further support the suggestion that the effect of wording differentially impacts engagement in keyword groups, rather than an interaction emerging due to the manipulation only affecting one group but not the other. Together, these findings suggest that ads with explicit suicide language are less likely to be clicked on than those with nonexplicit language when individuals are searching for low-risk keywords. The reverse was observed when searching for high-risk or help-seeking keywords (although the former did not reach significance), where ads with explicit language were more likely to be clicked on. This pattern of findings may partly contribute to the pattern of findings in the total conversion rate, in which analysis revealed 2-way interactions between low- and high-risk keywords and between low-risk and help-seeking keywords, suggesting that the effect of explicit wording in the ad differed among these groups. Exploratory analysis revealed that the interaction between low- and high-risk keywords may be driven by a higher total conversion rate when explicit wording is used with high-risk versus low-risk keywords, whereas there was no apparent difference between high-risk and low-risk keywords when nonexplicit wording was used. These findings suggest that having explicit wording has opposite effects, depending on the search terms used: explicit wording reduces the total conversion rate for individuals searching for low-risk keywords but increases the total conversion rate for those using high-risk keywords. There is no evidence to suggest that explicit or nonexplicit wording affects the total conversion rate when help-seeking keywords are used. These findings support both recommendations from lived experience advisors, both for and against the use of explicit wording, as perhaps both are appropriate for individuals in different cognitive states.

Overall, these findings suggest that individuals who search for help-seeking or high-risk suicide keywords respond more to ads and campaigns with explicit suicide wording, demonstrated by the higher click rate and total conversion rate, respectively. Alternatively stated, an ad campaign targeting individuals searching for high-risk keywords is likely to lead to more desirable behaviors if explicit wording is used. This may be because individuals may respond more strongly to a campaign that specifically targets their current situation. Given that the ad explicitly communicates and labels their current issue, the individuals may be more inclined to seek help. As the nonexplicit ad contained no indication that the campaign is for suicidality, the users may not have been certain that the webpage was able to meet their needs.

Furthermore, the high-risk search terms in this study excluded individuals who were explicitly searching for help for their suicidality. Thus, our findings suggest that explicitly naming the issue can improve help seeking for individuals at high risk of suicide but who may not be actively seeking help, as revealed in the total conversion rate. This finding is consistent with current practices in suicide prevention first aid (eg, applied suicide prevention skills training [9], where directly addressing and asking about suicide are strongly encouraged. This study demonstrated that the benefit of explicit wording for individuals in crisis is generalizable beyond suicide first aid and direct face-to-face communication. This finding may have implications for other forms of communication when addressing individuals at high risk of suicide.

The results also showed that individuals searching for low-risk suicide terms respond less to campaigns with explicit suicide wording. Since the low-risk keywords in this study were broad (eg, loneliness), many individuals searching for these terms may not have been suicidal—hence the lower click rate. However, despite being low, we still observed a click rate by individuals searching for low-risk keywords on the explicit campaign compared to the industry standard of 3.17% [13]. This suggests that individuals who may not be searching for explicit keywords may be experiencing suicidality and that targeting low-risk keywords is still beneficial. Conversely, we may have seen an elevated click rate for the low-risk, nonexplicit condition due to people clicking on the ad not realizing it was for individuals experiencing suicidality. Nevertheless, in the low-risk, nonexplicit condition, we still observed a conversion rate of over 20%, relative to the industry standard of 3.75%, suggesting that the landing page was still fulfilling a need. This may be because by using explicit suicide wording, we may alienate individuals who, for various reasons, may not recognize, identify, or acknowledge that their feelings are those of suicide. Thus, when visiting the page, their needs are met. However, one possibility is that some individuals searching for low-risk keywords are unaware that they are suicidal, and using the word “suicide” may help bring awareness to these underlying feelings [8]. Thus, explicit and nonexplicit keywords may have their own benefits; however, our findings suggest that overall, using nonexplicit keywords will reach more people. Although many people searching for low-risk keywords may not be experiencing suicidality, we must ensure that little effort is needed to access suicide-related resources and help, given that there are still individuals searching for low-risk keywords who are experiencing suicidality. Further research is needed to understand how best to tailor the ads to individuals searching for low-risk keywords.

The findings suggest that individuals experiencing suicidality and who could explicitly communicate it have higher engagement patterns with a campaign when the word “suicide” is used in the ad regardless of whether they are explicitly seeking help. However, for individuals experiencing general distress but not searching for suicide-specific terms, using explicit suicide wording leads to lower engagement with the campaign. Thus, in response to the finding that some lived experience advisors advocated for the explicit use of the word “suicide,” while others advocated against it, perhaps both are true for individuals in different cognitive states.

We recommend that the development and design of help-seeking prompts for suicide consider at what stage the individual is. If the prompt is intended for individuals with a lower risk of suicide, such as public media campaigns, then the use of the word “suicide” may decrease engagement; however, if the prompt is intended for individuals at high risk of suicide, whether they are or are not actively seeking help, then the use of the word “suicide” is likely to increase engagement. Furthermore, these findings suggest the need to codesign with a range of individuals who have experienced the spectrum of suicidality to understand their needs, the thought process, and how they speak about and internally conceptualize their distress and suicide to formulate different terms that promote help seeking and engagement.

Given that this pattern of finding has been found across 2 modalities (suicide first aid and internet ads), future research should seek to investigate the generalizability of these findings to other help-seeking prompts in suicide prevention, for example, signage at frequently used locations, safety planning app notifications, or the wording on the suicide hotline banner if individuals search for suicide-related terms. Further, future research should further understand what type of ad wording works for whom. For example, previous evidence has suggested that men and women respond differently to tailored ad campaigns [14]. Using Google Ads, we can further investigate what type of ad best engages men and women.

There was a low number of searches and engagement for means-specific keywords in this study. There may be several possibilities for this. First, these numbers may reflect true rates and only a few individuals were searching for these terms. Another possibility is that individuals at the planning stage do not primarily turn to search engines but may use other means of information seeking, as means selection has been found to be influenced by prior familiarity with the means itself [15]. Another possibility is that more individuals were searching for these terms but our current keyword list and Google’s function of generating permutations of the keyword list could not capture the range of search terms. This could be rectified by experimenting with keyword setting iterations in Google Ads to capture a wider variety of expressions. Finally, one possibility is that there are cultural differences in search behaviors. For example, a previous study found high incidences of method-related searches in Japan [16], suggesting that cultural differences may exist. Given that a key finding in previous research was that individuals who attempted suicide had searched for means-specific keywords [5], future research should investigate how individuals search for information regarding means and how we can best intervene at this stage.

Future studies may also investigate whether these findings generalize to other psychological or health domains. For example, an individual who is acutely depressed and can identify it may respond better to an ad for therapy explicitly communicating that this is for individuals experiencing depression. Conversely, an individual who may also be experiencing depressive symptoms but is either unfamiliar that they are experiencing depressive symptoms or is from a background where depression is strongly stigmatized may respond better to an ad without explicitly using the word “depression.” Future studies should carefully consider conversion actions, as we cannot measure directly whether an individual is processing the information presented to them, and thus, we use proxy measures common to the marketing field (eg, time spent on a page). Given the growing interest in online interventions, standardized methods for measuring engagements should be established.

### Limitations

The study has several limitations. One limitation is that due to resourcing constraints, there was a large difference in the number of participants in the 2 conditions. Furthermore, the data were collected at different times of the year, so there may be seasonal or cohort effects; future studies should run campaigns with explicit and nonexplicit wording at the same time. In addition, as research with this type of data is still in its infancy, future research should focus on understanding whether, when, and how search metrics should be normalized against variations in time. Another limitation is that we could only infer cognitive states from search results but did not measure suicidality directly. For example, a person may search for a high-risk keyword as part of a study but not be suicidal. Thus, there is added noise in the data.

### Strengths

Our study also has several strengths. The study was run nationwide, allowing us to sample the entire target population rather than just a specific subset. The components of this study, such as the ad wording, landing page, and keywords used to trigger the ad, were codesigned with individuals with lived and living experiences of suicide. Furthermore, by providing data on click rates and conversion rates, we obtained greater mechanistic insight into the findings for the total conversion rate.

### Conclusion

Our study demonstrates different engagement levels with an online suicide prevention campaign due to the word “suicide” in the search page ad. Future research should further explore what type of messaging works best for whom, and when paired with the flexibility of the advertising industry, we may be one step closer to ensuring that each person is met with a message that leads to the highest probability of them engaging services, using resources, or seeking help.

## Abbreviations

ad: advertisement
IRD: incidence rate difference
IRR: incidence rate ratio
